# The Phytochemical Potential for Brain Disease Therapy and the Possible Nanodelivery Solutions for Brain Access

**DOI:** 10.3389/fonc.2022.936054

**Published:** 2022-06-23

**Authors:** Yang Liu, Zhouchun Chen, Aijie Li, Runhan Liu, Haoying Yang, Xue Xia

**Affiliations:** ^1^ Henan-Macquarie University Joint Centre for Biomedical Innovation, School of Life Sciences, Henan University, Kaifeng, China; ^2^ Henan Key Laboratory of Brain Targeted Bio-nanomedicine, School of Life Sciences & School of Pharmacy, Henan University, Kaifeng, China

**Keywords:** phytochemicals, blood–brain barrier, brain diseases, nanodelivery, therapeutics development

## Abstract

Plant-derived phytochemicals have gifted humans with vast therapeutic potentials. Yet, the unique features of the blood–brain barrier significantly limit their accession to the target tissue and thus clinical translation in brain disease treatment. Herein, we explore the medicinal outcomes of both the rare examples of phytochemicals that can easily translocate across the blood–brain barrier and most of the phytochemicals that were reported with brain therapeutic effects, but a bizarre amount of dosage is required due to their chemical nature. Lastly, we offer the nanodelivery platform that is capable of optimizing the targeted delivery and application of the non-permeable phytochemicals as well as utilizing the permeable phytochemicals for boosting novel applications of nanodelivery toward brain therapies.

## Introduction

**Figure 1:**
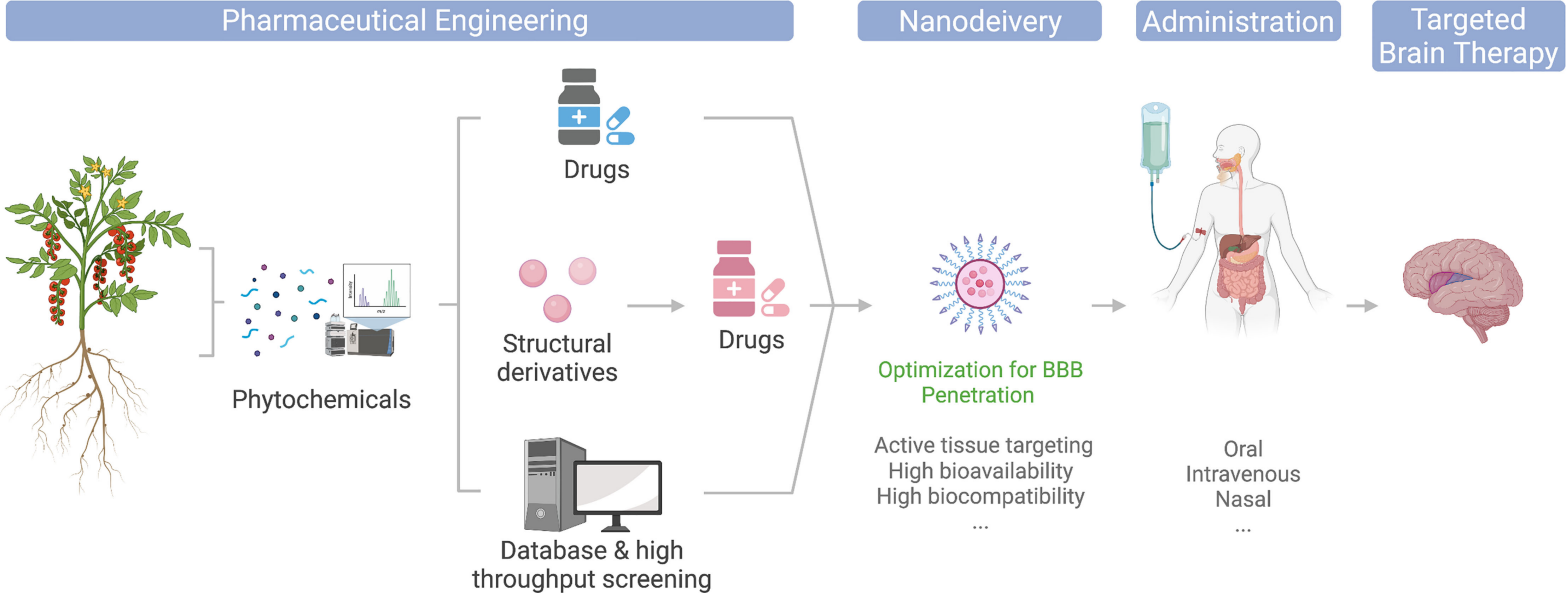
Illustration of phytochemicals undergoing different engineering approaches toward clinical and market-ready available drugs; nanodelivery platforms could potentially optimize their application for brain disease therapies.

The modern pharmaceutical industry was gifted by natural phytochemicals to develop popular medicinal compounds. Phytochemical is a term defining a wide range of natural compounds derived from plants (phyto) (1). Most celebrated examples include aspirin, artemisinin, and paclitaxel (2). Back in the very early days, humans discovered the therapeutic effects of herbs by either intentional or accidental intake and thus raised medicinal science from herbology. Modern pharmaceutical engineering has successfully applied plant-derived compounds to drug translation. Its general process involves isolating and purifying target phytochemicals followed by pharmacological capacity and pharmacodynamic evaluation in the laboratory and then transferring to druggability evaluation and drug development process. For better efficacy, some phytochemicals are subject to structural optimization and derivatized (**Figure 1**). Phytochemicals have also been incorporated into various small-molecule libraries for high-throughput drug screening and computer-based virtual drug screening. Their relatively complex structures also provide a new source for drug design and expansion of existing libraries. Moreover, the total synthesis of naturally produced phytochemicals with high economic values is also an important research area of modern organic chemistry, which focuses on reducing synthesis costs, improving yield and purity, and obtaining precursors that can be derivatized in various ways.

The entry of plant-derived drugs into the human body can involve different routes. Current main administration approaches include intravenous injection, oral administration, and nasal feeding (**Figure 1**). Since discoveries of the pharmacological properties of many phytochemicals were food sourced, they are generally equipped with relatively high gastric acid tolerance and intestinal absorption and thus make their oral administration possible (3). However, due to their low targeted organ accumulation, and the existence of the blood–brain barrier (BBB), the bioavailability of orally administrated phytochemicals is generally low. Thus, a large number of phytochemical candidates remained in their laboratory evaluation stage, or became healthcare products, for their unsatisfying direct therapeutic effects (3, 4). Intravenous administration avoids drug loss in the gastrointestinal tract and thus increase drug concentrations in the blood, yet it is still limited for brain targeting. The booming development of nanomedicine has provided a whole collection of cutting-edge brain delivery platforms with promising results in animal experiments (5–7), and some even made to clinical approval (**Figure 1**). In addition, nasal administration is also considered a potential strategy to increase drug accumulation in the brain. Combining nasal administration with special drug formulations, such as lipid-soluble solvents, could ideally reduce the drug loss in blood circulation and enter the brain through the olfactory bulb easily. However, due to the huge difference of the olfactory bulbs between mice and human in terms of their size proportion to the brain, many drugs remain non-applicable for human (8). In regard to this, nanodelivery formulations are also expected to improve the BBB translocation efficiency of nasogastric administration (**Figure 1**).

Current clinical applications of phytochemicals are faced with challenges for sufficient targeted organ accumulation, for which much clearer mechanisms of their functional pathological mechanisms must be further investigated. Many phytochemicals were found to play a role in a wide collection of therapies, yet their molecular functioning mechanisms remain elusive. Differing from healthcare products, a clinical drug requires a clear understanding of its pathological interaction and metabolism, as well as a defined dose, efficacy, and clear indications. Further studies on its functioning mechanisms, especially in combination with structural biology and computational drug design, may provide more novel drug candidates from phytochemical sources to cope with the limited screening libraries and the relatively simple structures in the current pharmaceutical screening banks.

The current review provides an update and evaluation of BBB-permeable and non-permeable phytochemicals with existing knowledge of their therapeutic effects toward brain treatment, particularly introducing the nanodelivery platform for maximizing the utility of phytochemicals in brain therapies (**Figure 1**).

## Brain Diseases and the BBB

**Figure 2:**
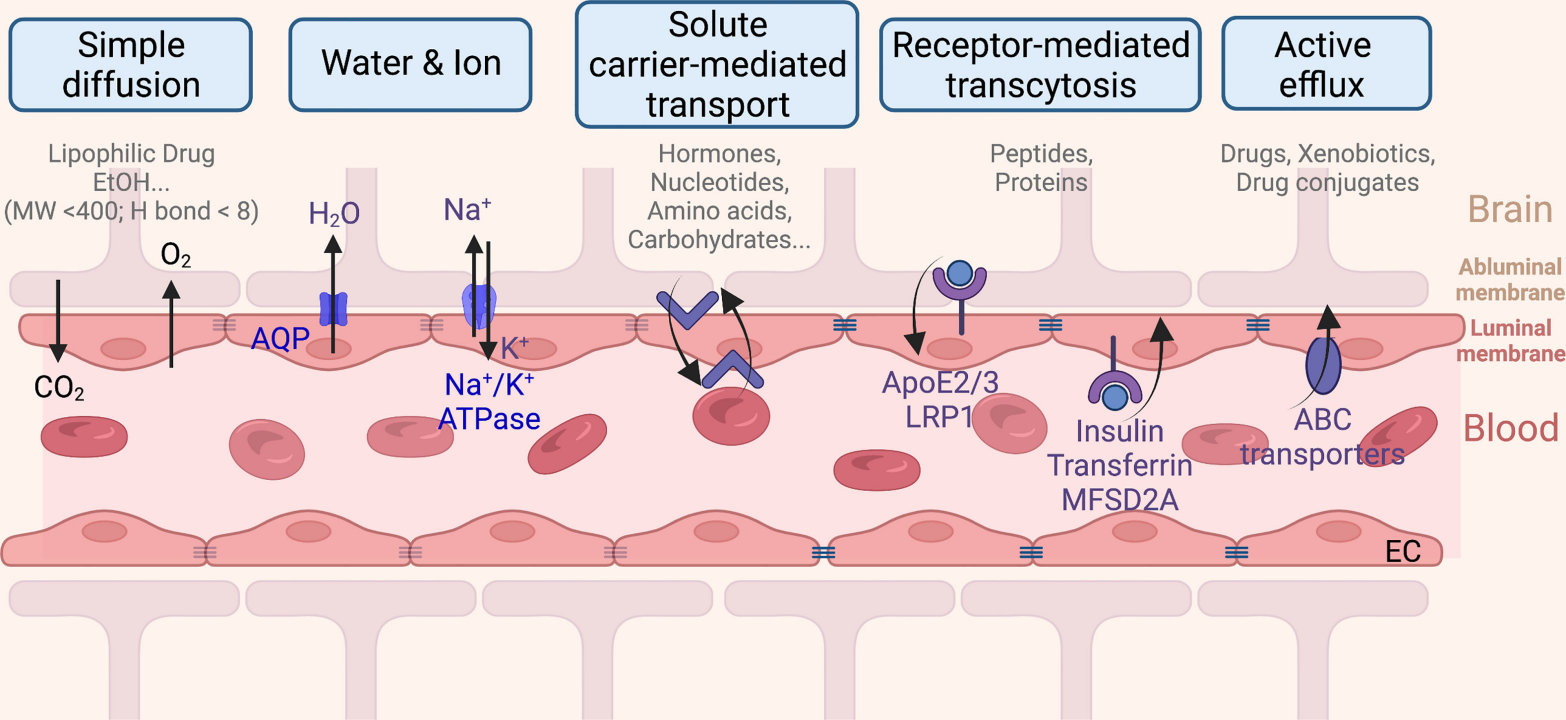
The blood–brain barrier monitored transportation from the capillary endothelium to the brain.

The BBB is a dynamic multicellular layer separating the peripheral blood circulation and the central nervous system (CNS). It is constituted by endothelial cells (ECs) of brain micro-vessels and supported by astrocytes, pericytes, and extracellular matrix components (9). The BBB is critical for maintaining the proper environment, nutrients, and oxygen to neuronal functions, as well as limiting entries of toxins, pathogens, or cells from the blood into the brain. Brain ECs (BECs) have a complex molecular transporting system and have their unique features in comparison to ECs in other tissue. The built-in continuous intercellular tight junctions (TJs) of BECs (**Figure 2**) lack fenestrations and undergo an extremely low rate of transcytosis, thus greatly limiting both paracellular and transcellular molecular exchanges (9) of nearly 98% of generic molecules, leaving only some lipid-soluble small molecules (i.e., molecular weight < 400 Da or containing less than eight hydrogen bonds) (10) that are able to cross (11). Therefore, delivery of nutrients is limited only to be mediated *via* a series of specific transporters that allow selective cargoes (i.e., nutrients, ions, few peptides, proteins, and fluid) across the BBB (**Figure 2**) (12). For instance, BBB transport of water and ions is mainly mediated by aquaporin (AQP) and the abluminal sodium pump (Na^+^-, K^+^-ATPase), respectively. Ethanol and other small lipophilic molecules can cross the BBB through simple diffusion. Metabolites, nucleotides, and other substrates can be transported by solute carrier-mediated transport with concentration gradient, while peptides and proteins are mainly transported by receptor-mediated transcytosis (RMT) (13). In addition, a few immune cells can infiltrate into the healthy CNS across the BBB due to the BECs having a relatively low expression of immune cell recognition markers, i.e., leukocyte adhesion molecules (14). Therefore, the immune surveillance system in CNS is also unique. Taken together, all these differences limited our further understanding of brain diseases.

Emerging studies revealed that the BBB has a very close link to brain pathological changes. The breakdown of intact BBB by tissue damage, injury, pathogen, or pathological molecular events disturbs the microenvironment and/or transport processes and can cause a series of brain diseases. Debris from damaged brain must also be cleared by immune cells. Moreover, the subsequently increased immune cell incursion and molecule flux dysregulations happen when the BBB breaks down, and immune surveillance and effector responses to brain infections may trigger inflammation and multiple diseases (15). Ample examples of neurodegenerative disease development are linked to breakdown of the BBB, during which multiple pathological changes are involved, such as increased BBB permeability, microbleeds, impaired p-glycoprotein function, compromised glucose transport, CNS leukocyte infiltration, capillary leakages, pericyte degeneration, endothelial degeneration, abnormal angiogenesis, and other molecular pathological changes (12).

## Glioblastoma Therapy and BBB Permeability

Glioblastoma is the most frequent and aggressive malignant primary brain tumor of the central nervous system in adults (16). Its extremely poor prognosis left patients with a median survival less than 15 months (17, 18). Current treatments for GBM include chemotherapy, radiotherapy, and surgery. Yet, the chemotherapy compounds for GBM therapy are extremely limited, with temozolomide (TMZ) being the only orally administered drug approved by the US Food and Drug Administration (FDA) (19). TMZ is a DNA-alkylating agent that mediates the breakdown of the DNA double strand and eventually leads to cell death. Being the only frontline treatment against GBM, TMZ was administered in GBM xenograft mouse models *via* different routes. Intraperitoneal injection at 50 mg/kg for three times across a 2-week period (20) or a single orally administered TMZ treatment at 600 mg/kg (21) both showed ideal outcomes in mouse GBM xenograft models. The development of an instructive dosage of chemotherapy drugs like TMZ (sourced from Medscape and Mayo Clinic) requires extensive preclinical (animal models) and clinical trials. More importantly, it is the active amount of drugs at the site (i.e., brain tumor tissue) that is of greater importance for GBM therapy than the given dosage. For instance, a study using the intracerebral microdialysis determined the neuropharmacokinetics of TMZ in the brain interstitium after oral administration (150 mg/m^2^ in human) and reported a mean peak TMZ concentration in the brain of 0.6 ± 0.3 μg/ml after about 2 h (22). However, our understanding of the at-site active concentration remains mostly elusive for current clinical-approved drugs (23) and future potential drugs (24) and thus require much further investigation.

Unfortunately, as the standard-of-care chemotherapy drug for GBM, TMZ is faced with significant drug resistance challenges, with at least 50% of TMZ-treated patients failing to respond (25). Therefore, the search for new chemotherapy drugs with better outcomes and fewer side effects is still in the chase. Phytochemicals come in the hunt as a natural product and thus attracted the most attention. Application of phytochemicals for cancer therapy was suggested in clinical tests for various cancer types. Examples include *Allium sativum* for the treatment of inoperable colorectal, liver, or pancreatic cancer [more clinical cases were excellently reviewed by Hosseini and Ghorbani (26)]. Nevertheless, as stated above, the BBB restricted the brain access for most external compounds. To be more specific, the BBB permeation decreases 100-fold when the molecular weight of a drug increased from 150 to 450 Da (27), and with each pair of hydrogen bonds on the solute, a 10-fold decrease is reported in its *in vivo* BBB transport (28). Thusly, the generally more complex chemical structures of phytochemicals are likely to be impermeable to the BBB and restricted their subsequent therapeutical use toward brain diseases, leaving rare examples for phytochemical treatment toward GBM. To this end, one study employed quercetin and demonstrated that at a dosage of 20 mg/kg/day for 14 days, quercetin was able to sensitize GBM to t-AUCB by dual inhibition of Hsp27 and COX-2 both *in vitro* and *in vivo* (29). Meanwhile, another group demonstrated that *Angelica sinensis* showed an anti-GBM effect, although a higher dose (500 mg/kg/day) was exercised for the study (30). In the meantime, other investigations targeting GBMs are primarily performed at the *in vitro* level (31). However, the silver lining is that phytochemicals indeed demonstrated neuroprotective capacities while targeting other brain diseases (neurodegenerative diseases), including Alzheimer’s disease (AD) and Parkinson’s disease (PD) (32), which could be potentially referenced by future GBM therapy studies.

## Limited Numbers of Phytochemicals Are BBB Permeable for Brain Diseases

Current therapeutic applications of phytochemicals toward brain diseases are heavily relying on their permeability across the BBB, and their corresponding therapeutical dosage remains controversial. As discussed above, the administered dosage of a drug is only one of the instructions in determining whether the drug has high therapeutic efficacy. However, with the very limited information available, we try to gather more instructions and hereby report examples of phytochemicals that are more likely or unlikely to have BBB permeability when their administered dosage was lower or higher than 10 mg/kg/day, respectively. This dosage measurement only plays a suggested guidance and irrespective of the administered routes, as all current routes suffer from significant loss during blood circulation or gastric consumption. Among known candidates, polyphenols (33) and flavonoids (34) are the most well-studied subtypes of phytochemicals and were considered to have neuroprotective effects and broad-spectrum disease treatment effects including cardiovascular diseases, metabolic syndrome, and cancer. Unfortunately, the translocation mechanism of these defined phytochemicals that are easier to pass through the BBB remains elusive.

Moreover, understanding of the underlying functional mechanisms of bioactive phytochemicals toward brain disease therapies remains limited. Some rare examples with scarce clues include luteolin, the abundant flavonoid in the leaves of *Capsicum annuum* (35, 36); peripherally administered luteolin (5 and 10 mg/kg/day by gavage for 4 weeks) was able to significantly abolish amyloid-β (Aβ) deposition, glycogen synthase-3 (GSK-3) activation, phospho-tau, and pro-inflammatory cytokines in a traumatic brain injury-induced mouse model (37). Luteolin also promotes the translocation of nuclear erythroid 3-related factor 2 (Nrf2) to the nucleus in both *in vivo* and *in vitro* conditions, and the neuroprotective effects of luteolin in traumatic brain injury were suggested to be through the activation of the Nrf2–ARE pathway (38). Moreover, icariin (ICA), a natural compound derived from *Herba Epimedii* (39), showed neuroprotective effects on dopaminergic neurons in a PD mouse model, and the potential mechanisms might be related to phosphatidylinositol-3-kinase (PI3K)/protein kinase B (Akt) and mitogen-activated protein kinase kinase (MEK)/extracellular signal-regulated kinase (ERK) pathways (40). Application of icariin at 2 or 6 mg/kg/day for 4 months in Sprague-Dawley rats is able to upregulate autophagy-related proteins in the cortex and hippocampus of aged rats (41), while a higher gavaging dosage at 80 mg/kg/day for 3 months in a senescence-accelerated mouse-prone 8 (SAMP8) model exhibited a reduced number of senescence cells, neuronal loss, and the expression of autophagy-related proteins (42). In the meantime, administration of 4-*O*-methylhonokiol, isolated from *Magnolia officinalis*, elevated lipopolysaccharide-induced neuroinflammation, amyloidogenesis, and memory impairment *via* inhibition of nuclear factor-kappaB (NF-κB) signaling pathway in *in vitro* and *in vivo* models in a dose-dependent manner (43). Swedish Aβ PP AD model mice pretreated with 4-O-methylhonokiol (1 mg/kg/day) for 3 months showed recovered memory impairment and inhibited neuronal cell death by limiting the expression level as well as the activity of beta-site Aβ PP cleaving enzyme (BACE-1) (44). Another study using the same dosage on presenilin 2 mutant mice revealed its capacity of reducing TNF-α, IL-1β, reactive oxygen species (ROS), and nitric oxide (NO) in neurons and ERK pathway activation in cultured astrocytes. Moreover, withanamides of the *Withania somnifera* L. fruit extract were reported to be equipped with lipid peroxidation inhibitory capacity. Intraperitoneal injection (i.p. injection of 5 mg/kg for once) of withanamides in mice confirmed their BBB permeability (45). Selective activation of p53 by withanamides in tumor cells results in limited growth and apoptosis. Moreover, the cellular toxicity is addressed *via* inhibition of mitochondrial respiration (46) and DNA damage (47), evidenced by the increase in γ-H2AX and number of cells expressing the phosphorylated form (48).

It is very common for phytochemicals to have significant therapeutic effects reported extensively, but their underlying working mechanisms rest unclear. Obovatol, a biphenolic compound from *Magnolia obovate*, has neuroprotective capabilities toward neuroinflammation (49), and the administration of obovatol (1 mg/kg/day for 3 months) with mouse-expressing mutant human amyloid precursor proteins significantly improved cognitive functions (50). At a dosage of 10 mg/kg/day *via* i.p. for 4 days, obovatol showed neuroinflammation inhibition in lipopolysaccharide-induced neuroinflammation in C57BL/6 mice (49).

Another subgroup of examples is everyday-accessible phytochemicals, including caffeine (1,3,7-trimethylxanthine), the dominant physiologically active compound in coffee beans and many other soft drinks (51). Classified as a purine alkaloid, caffeine is able to translocate across the BBB (52), and several clinical studies have correlated its consumption with lower risk (30%–38%) of PD (51). Another polyphenol, chlorogenic acid, found in green coffee beans (70–350 mg per cup of coffee) and in some other fruits, vegetables, olive oil, wine, and tea (51), is also demonstrated to be able to cross the BBB (53), and its neuroprotective effects are linked mainly due to its ability to reduce oxidative stress. Vardi et al. (54) demonstrated that chlorogenic acid is able to protect the rat brain cerebellum from oxidative damage by inhibiting lipid peroxidation, augmented the antioxidant defense system, and prevented mitochondrial dysfunction and DNA fragmentation.

## BBB Is a Challenging Boundary for Most Phytochemical Drugs With Great Brain Therapeutic Potentials

Unfortunately, various phytochemicals failed their further application in brain diseases; although many studies suggested these phytochemicals are beneficial to brain disease treatment, their successful clinical translations are rare. The therapeutic potential of administered phytochemicals is significantly limited due to the presence of the BBB, restricting accession for most phytochemicals and thus their brain disease therapeutic use. To obtain sufficient brain accumulation of the phytochemical drugs, the incredible high-dose administration thus remains a biocompatibility concern.

Curcumin, a hydrophobic polyphenol extracted from the dried rhizomes of *Curcuma longa* L., is reportedly able to reduce cytokine production, inhibiting NF-κB signaling pathway, as well as suppressing neuroinflammation in amyloid precursor protein and presenilin 1 (PS1) double-transgenic (APP/PS1) mice (i.p. injection at 150 mg/kg/day for 4 weeks) (55). Other studies also demonstrated the beneficial effects of curcumin in preventing Aβ_42_ oligomer formation and disaggregation of the formed fibrils and thus benefitting AD outcomes (56). Despite its possession of crucial neuroprotective properties, using curcumin in neurodegenerative diseases and brain tumor therapies is still limited because of its poor brain bioavailability owing to poor absorption and stability at physiological pH, high rate of metabolism, rapid systemic elimination, and limited BBB permeation (57).

Salidroside from *Rhodiola rosea* (58) is able to translocate across the BBB (59) and enhance the cognitive recovery of AD rats by regulating the expressions of thioredoxin, thioredoxin-interacting proteins, and NF-κB pathway proteins (60). Salidroside shows therapeutic effects at a dosage of 50 mg/kg/day for 3 months in a senescence-accelerated mouse model with reduced Aβ_1-42_ deposition and microglial activation (61). Meanwhile, a 25-mg/kg/day for 8-week gavaging treatment profoundly improved cognition dysfunction in aged mice and alleviated neuronal degeneration in the aging mice hippocampal CA1 region. Further evaluation of the treated mice suggested that salidroside promotes telomerase reverse transcriptase expression *via* the PI3K/Akt pathway (62).

In the meantime, geniposide, an iridoid glucoside, purified from *Gardenia jasminoides* Ellis, was reported able to suppress the receptor for advanced glycation end product (RAGE)-related signaling such as ERK and NF-κB (63) and production of tumor necrosis factor-α (TNF-α) cerebral Aβ accumulation in an mPrP-APPswe/PS1dE9 AD transgenic mouse model at 25 mg/kg/day for 3 months (63). For erinacines from *Hericium erinaceus* (64), a 108-mg/kg/day for 12-week feeding program of erinacine was proved to be sufficient for improving the learning and memory capacity in a 3-month-old senescence-accelerated mouse model (65). A dosage of 300 mg/kg/day for 30 days was also able to decrease Aβ plaque burden in an AD mouse model (66). Paeoniflorin is highly water soluble and impermeable for the BBB (67); it exhibits neuroprotective effects in APP/PS1 mice *via* inhibiting neuroinflammation mediated by the GSK-3β and NF-κB signaling pathways and nucleotide-binding domain-like receptor protein 3 (NLRP3) inflammasome (68). Another polyphenol, resveratrol, derived from wine grapes, have potent antioxidant, anti-inflammatory, antiaging, and neuroprotective activities. Baicalein (5,6,7-trihydroxyflavone; C_15_H_10_O_5_), wogonin, and baicalin are flavonoid compounds isolated mostly from the roots of *Scutellaria baicalensis* Georgi (Labiatae) (69) that is able to penetrate the BBB within 30 min. Both baicalein and baicalin have killing effects to a collection of human tumor cells (70), as well as inhibiting tumor growth *in vivo* (71). The tumor-inhibiting effect is *via* inducing apoptosis, triggering autophagy, arresting the cell cycle, and inhibiting 12-lipoxygenase and metastasis suppression (72). A dose-dependent oral administration of baicalein (400 mg/kg/day for 42 days) increased the number of TH^+^ neurons in rotenone-induced PD model rats (73). Additionally, epigallocatechin-3-gallate (EGCG), the major flavanol found in green tea (*Camellia sinensis*), recovered the learning ability of brain-accelerated senescence model mice (SAMP10) that ingested EGCG (20 mg/kg/day for 2 weeks) (74), showing its beneficial effects on cognitive dysfunctions; however, its high dosage still limits its clinical application even though the organic anion-transporting polypeptides (SLC21A3) on the BBB facilitate EGCG to penetrate into the brain (75, 76).

Another most celebrated phytochemical that is not permeable to BBB is the phytocannabinoid derivatives (77). The most famous and responsible for the pharmacological activity compounds of *Cannabis sativa* L. are the psychoactive Δ^9^-tetrahydrocannabinol (THC) and cannabidiol (CBD) (78). Cannabis constituents THC and CBD also inhibit T-helper type 1 (Th1) cytokines and/or promote an *in vitro* and *in vivo* Th2 immune response. Their multifunction results from the affinity of these compounds predominantly for the receptors of the endocannabinoid system (the cannabinoid receptor type 1 (CB1), type two (CB2), and the G protein-coupled receptor 55 (GPR55)) but also for peroxisome proliferator-activated receptor (PPAR), glycine receptors, serotonin receptors (5-HT), transient receptor potential channels (TRP), and GPR, opioid receptors (79).

The functional elements of phytochemical identification/extraction promoted the development of analytical chemistry. In modern pharmaceutical industry, the screen library encountered the strike of lack of structural complexity, which means most of the easy-to-synthesize compounds have been engaged into the screening bank and no longer sufficient for further high-throughput screening and need to update with more structure complexity. The phytochemical derivatives may be a rich resource for this revolution.

The advantages of phytochemical derivatives also rest in many aspects such as the low cost of biosynthesis and the relatively high biological reactivity due to is endogeneity. In recent years, the *de novo* chemical synthesis for therapeutic phytochemicals has aimed for lower costs and higher purity is emerging as an important study in synthetic chemistry. Therefore, it is likely that future pharmaceutical sciences will continually gain insights from phytochemicals and their derivatives.

In the meantime, insightful studies have also provided a collection of databases for phytochemicals; renowned examples include Dr. Duke’s Phytochemical and Ethnobotanical Databases at NAL (https://phytochem.nal.usda.gov/phytochem/search) and Search Phytochemical Databases (leffingwell.com) (80), while the US Food & Drug Administration also established its approved phytochemical drugs in its database at https://www.fda.gov/drugs/development-approval-process-drugs/drug-approvals-and-databases.

## Nanodelivery Systems Provide Solutions for Phytochemical Brain Therapies

The above discussions have denoted the BBB-permeable or -impermeable phytochemicals, which are entitled with numerus therapeutic potentials. However, their *in vivo* or potential clinical application in brain treatment is still restricted by their bioavailability across the BBB. To this end, nanoparticles could rise as a potent delivery platform, addressing a series of issues in regard to the clinical applications of phytochemicals, including solubility, stability, target specificity, effective accumulation, and passing through the BBB.

Nanoparticles are termed as materials or structures in a nanometer scale. Their size allows the nanoparticles to have potential access to cell barriers (81). Therapeutical nanoparticles can be generally categorized into liposomes, polymers, dendrimers, micelles, engineered biomaterials, and inorganic nanocarriers (5). Different formulas of nanoparticles each have their own pros and cons when it comes to function as drug carriers (82); however, it is the facile physical and chemical characterization that can be modified upon requirement that satisfies the optimization of phytochemicals *in vivo* or even clinical application toward brain diseases. Moreover, the targeted delivery and release capacity of nanoparticles could potentially lower the drug dosage *via* oral or i.p. administration substantially.

Loading of its cargo usually employs the intrinsic properties of the targeted drug formula, such as electronic charges or water solubility. Upon administration, release-at-site is also required for a successful nanomedicine (83). To achieve efficient controlled release, specific conditional stimulus-responsive mechanisms are implemented. Outstanding examples include redox-sensitive, pH-sensitive, temperature-sensitive, or aided by external stimuli such as ultrasonic, magnetic, or laser (84–86). The unique lower pH and redox-active brain tumor environment for instance are ideal for redox-sensitive or acidic triggered tumor site release of nanoparticle cargos.

**Figure 3:**
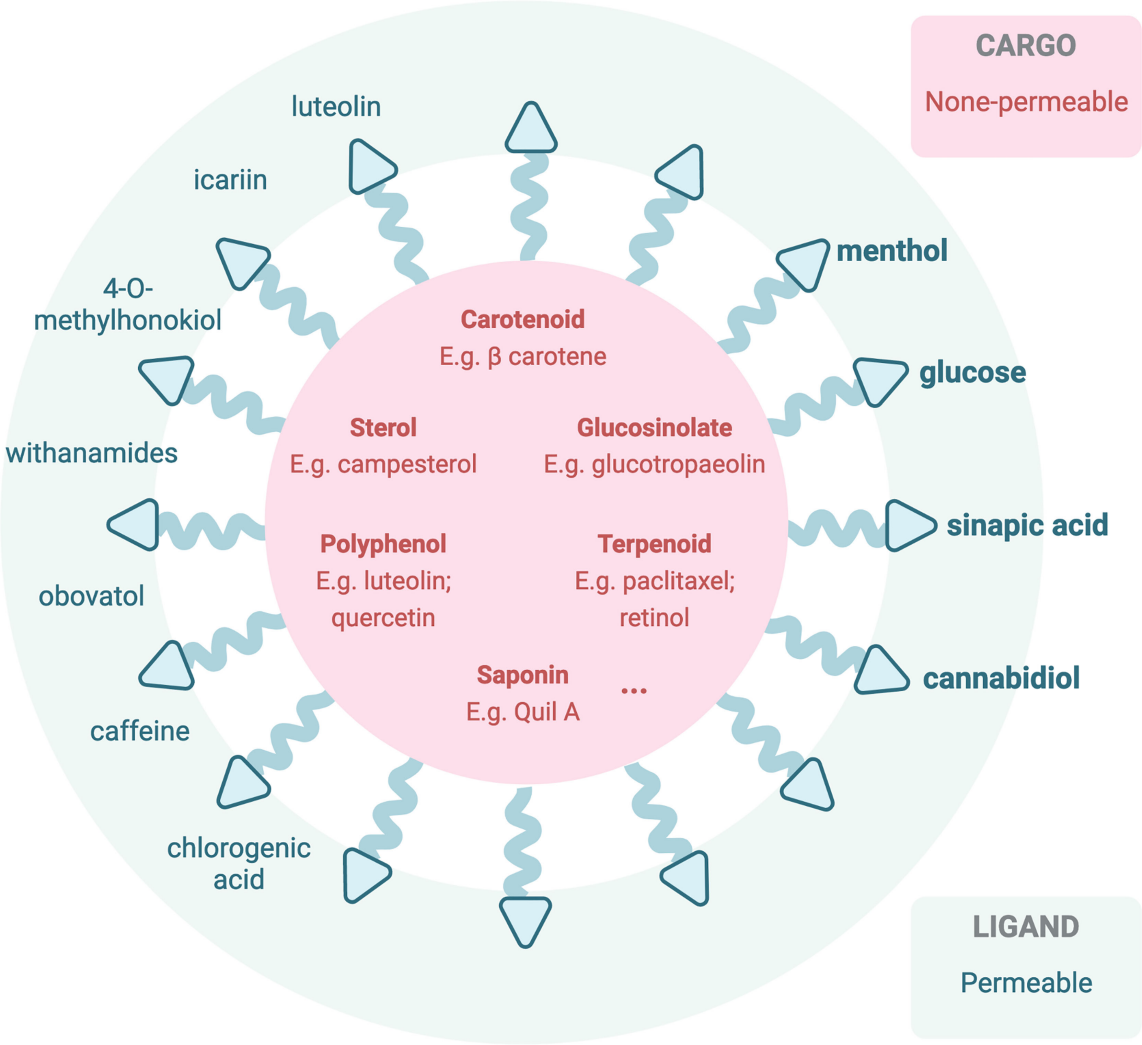
Illustration of current or potentially applicable nanoparticles loaded with therapeutic active phytochemicals (impermeable to the BBB) (90, 91) or decorated with BBB-permeable phytochemicals for *in vivo* brain therapeutic uses. Bolded phytochemicals are ones that have been reported in previous studies based on PubMed database (92–95).

In employing nanoparticles facilitating phytochemical brain therapies, different approaches were developed by researchers. The first technique is loading nanodelivery systems with phyto-bioactive compounds that have been confirmed to have a potential to modulate oxidative stress and inflammation known to be important players in brain-associated pathological conditions. Over several recent years, numerous convincing studies have reported that phytochemical-loaded nanocarriers can be highly effective in counteracting brain/CNS-related diseases (87) including neurodegenerative disorders, rheumatoid arthritis, and glioblastoma (88, 89). This approach aims to elevate the BBB permeability of the active phytochemicals, which are listed in Section 4 as BBB-impermeable ones (**Figure 3**). Quite surprisingly, despite the widely undergoing or even finished clinical trials [extensively reviewed by Chelliah and colleagues, Table 8 (87)], the actual application of the therapeutic active phytochemicals reviewed in Section 5 is still limited due to the presence of the BBB, and only a few turned to nanocarriers for *in vivo* brain tumor treatment. In one study, curcumin-loaded poly(lactic-co-glycolic acid) at 25 mg/kg (96) and tripalmitin-oleic acid lipid (5 days/week at 150 mg/kg) (97) nanoparticles were used for brain cancer treatment in an *in vivo* model. Resveratrol crosses the BBB and increased the antioxidant activity in AD rats (98). In a Sprague-Dawley rat model, researchers have employed spherical nanoparticles loaded with resveratrol for the treatment of intracerebral hemorrhage injury whose dosage was reduced to 5 mg/kg for once (99), in comparison to intragastrical administration of pristine resveratrol at 20 mg/kg/day for 42 days in levodopa-induced dyskinesia treatment (100). Another study employed resveratrol-loaded polyethylene glycol-polylactic acid nanoparticles in C6 orthotopic rats and U87MG-xenograft mice at an equivalent of 10 mg/kg *via* intravenous injection every second day for six times, significantly reduced the tumor volume, and prolonged life expectancy (101).

In the meantime, another pathway across the BBB can be modified *via* transporter uptake or other mechanisms to be accessible to the brain (76) (**Figure 3**). Transporters including the ATP-binding cassette (ABC) transporters and the solute carrier (SLC) transporters expressed at the luminal side of the BBB, *via* active efflux from the endothelium into the blood, play pivotal roles in the bioavailability and disposition of most drugs (76). Phytochemicals, especially ones with a smaller molecular size, that may be able to cross the BBB *via* transporter-mediated transcytosis, in this case reported in Section 4 as BBB-permeable phytochemicals, could potentially be employed to decorate the nanocarriers for transcytosis. One of the common examples is glucose, a common carbohydrate, which can be employed (102) to mediate nanoparticle transcytosis across the BBB *via* Glut-1 transporter, which is highly expressed on the luminal side of the BBB (94).

Other translocating mechanisms employing the unique effect of the BBB upon receiving the phytochemicals are also explored by researchers (**Figure 2**). For instance, menthol could enhance nanoparticles to translocate across the BBB (95), attributing to its significant enhancement on cell membrane fluidity and thus a decrease in membrane potential (103), during which process menthol enhances transport by its disassembly effect on tight-junction integrity (103). The functionalized menthol NP enabled nanoparticles across the brain capillary endothelial cell monolayer by promoting their internalization and BBB disruption (95). Another small molecule, sinapic acid (SA), extracted from mustard (93) and cannabidiol (104), was selected as a novel bioinspired BBB-permeable ligand for efficient drug delivery into the brain. However, its mediating transporter(s) across the BBB remains unclear by far.

Moreover, phytochemicals themselves could also be functionalized into part of the nanoparticle to facilitate their uptake across the BBB and, upon release inside the brain microenvironment, utilize the therapeutical nature of the phytochemical for treatment. One study disguised EGCG by functionalized EGCG; EGCG nanoparticles (NPs) were developed *via* a one-step polyphenolic condensation reaction (105).

## Conclusion

This review discusses the unique features of the blood–brain barrier and how it interferes with phytochemical application into brain diseases. Most phytochemicals show poor blood–brain barrier penetration capacity, yet ideal brain therapy potentials are reported. A possible nanodelivery platform was raised to optimize the utilization of phytochemicals, both permeable and non-permeable across the blood–brain barrier, thus offering a new avenue for phytochemicals toward brain disease clinical applications.

## Author Contributions

Conceptualization, XX, YL; writing, ZC, AL, RL; writing—review and editing, XX, YL; visualization, ZC, AL, HY; supervision, XX and YL. All authors contributed to the article and approved the submitted version.

## Funding

This work was supported by the National Natural Science Foundation of China (Grant No. 32001432) and the Fellowship of China Postdoctoral Science Foundation (Grant No. 2021M690903) and Department of Science and Technology Henan Province (202102110478).

## Conflict of Interest

The authors declare that the research was conducted in the absence of any commercial or financial relationships that could be construed as a potential conflict of interest.

## Publisher’s Note

All claims expressed in this article are solely those of the authors and do not necessarily represent those of their affiliated organizations, or those of the publisher, the editors and the reviewers. Any product that may be evaluated in this article, or claim that may be made by its manufacturer, is not guaranteed or endorsed by the publisher.
